# Customisation and validation of a low-volume plasma renin activity immunoassay: Enabling of regulatory compliant determination in paediatric trials

**DOI:** 10.1016/j.plabm.2019.e00144

**Published:** 2019-11-12

**Authors:** F.K. Suessenbach, J. Tins, B.B. Burckhardt, the LENA Consortium

**Affiliations:** Institute of Clinical Pharmacy and Pharmacotherapy, Heinrich Heine University, Universitaetsstr. 1, 40225, Dusseldorf, Germany

**Keywords:** Plasma renin activity, Paediatric, Immunoassay, Pharmacodynamic, Low-volume, Validation

## Abstract

**Objectives:**

Investigations of plasma renin activity (PRA) in children are urgently required. Small-volume, regulatory guideline compliant, bioanalytical assays tailored for paediatric application could facilitate to overcome this hurdle. Ethical constraints given e.g. by the European Medicines Agency need to be addressed and reliable data generation in line with Good Clinical Laboratory Practice must be ensured.

**Methods:**

A PRA enzyme-linked immunosorbent assay (ELISA) was tailored for paediatric application and validated in the context of the U.S. Food and Drug Administration bioanalytical guideline. Performance verification of the assay was conducted by participation in an interlaboratory ring test, evaluation of incurred sample reanalysis and an application-orientated approach in children.

**Results:**

A five-fold reduction of required plasma volume to 100 μL was achieved without limiting the calibration range. Between-run accuracy and precision varied no more than 5.0% and 6.3%, respectively. No substantial matrix effect was detected and the inter-run precision for parallelism was 11.1%. Stability experiments approved the freeze-thaw stability, short-term stability as well as 37 weeks of long-term stability. The assay successfully participated in the interlaboratory ring test, showing non-inferiority regarding radioimmunoassay (RIA). Moreover, PRA in plasma samples of neonates was successfully determined. Conducted incurred sample reanalysis confirmed the comparability and reliability of the assay with regard to international regulatory bioanalytical guidelines.

**Conclusion:**

A fit-for-purpose PRA ELISA characterised by low-volume application was successfully established, indicating non-inferiority regarding commonly applied RIAs. Reliability of the regulatory-compliant PRA assay was proven by participation in an interlaboratory ring test and its application in a paediatric population.

## Highlights

• A small-volume PRA ELISA was successfully validated regarding FDA guideline.
• Performance of the presented ELISA showed non-inferiority to corresponding RIAs.
• Substantial reduction in plasma volume enables application in vulnerable population.
• Application in diseased children and incurred sample reanalysis successfully proven.

## Abbreviations

ACE: Angiotensin-converting enzyme
AngI: Angiotensin I
CV: Coefficient of variation
CVD: Cardiovascular disease
CS: Calibration standard
ELISA: Enzyme-linked immunosorbent assay
EMA: European Medicines Agency
FDA: Food and Drug Administration
GCLP: Good Clinical Laboratory Practice
HF: Heart failure
HRP: Horse radish peroxidase
LBA: Ligand binding assay
LLOQ: Lower Limit of Quantification
OD: Optical density
PRA: Plasma renin activity
PD: Pharmacodynamic
PMSF: Phenylmethylsulfonyl fluoride
PK: Pharmacokinetic
QC: Quality control
RAAS: Renin-angiotensin-aldosterone system
RE: Relative error
RIA: Radioimmunoassay
RT: Room temperature
SD: Standard deviation
TMB: Tetramethylbenzidine
ULOQ: Upper Limit of Quantification

## 1 Introduction

Cardiovascular diseases (CVDs) are a leading cause of death in the Western world [1]. Among these, heart failure (HF) is responsible for high rates of hospitalisation, health-care-costs and mortality in adults [2]. HF with preserved ejection fraction is characterised by insufficient cardiac output and is thus associated with an undersupply of metabolising tissues with oxygen, leading to oedema, respiratory distress, fatigue and weakness as clinical symptoms [3]. It had been shown that the renin-angiotensin-aldosterone system (RAAS) is actively involved in the development and prognosis of HF [4].

The RAAS is characterised by an enzymatic cascade, beginning with the precursor peptide angiotensinogen, which is cleaved by the endopeptidase renin to angiotensin I (AngI). Subsequently, AngI is processed via the angiotensin-converting enzyme (ACE) to the bioactive peptide angiotensin II and corresponding degradation products (angiotensin III, angiotensin IV, angioprotectin, and angiotensin A) [5]. The RAAS has a major impact on homeostasis, blood pressure as well as salt and water balance; therefore, it affects the prognosis and aetiology of HF. Among the humoral parameters involved in the RAAS, plasma renin activity (PRA) is commonly utilised for evaluating metabolic dysfunctions (e.g. PRA-aldosterone ratio for the diagnosis of primary hyperaldosteronism, a cause of hypertension and trigger for diverse CVDs, including HF) [6]. Corresponding reference values for the classification of disease severity are well established in adults, while reliable data in children are lacking. Notably, the lack of reliable data for children impedes the development of efficient treatment options and subsequently puts children in jeopardy [7]. Moreover, high rates of off-label and unlicensed drug use in the paediatric population indicate a gap in current knowledge of PRA and further RAAS-related parameters in the very youngest [8].

To overcome this hurdle, sophisticated investigation of PRA values in children suffering from HF are urgently required. Consequently, small volume, regulatory guideline [9] compliant bioanalytical assays tailored for paediatric application are necessary. These assays need to address the ethical constraints given e.g. by the European Medicines Agency (EMA) (e.g. restricted withdrawable blood volumes in children [11]), as well as to ensure reliable data generation in line with Good Clinical Laboratory Practice (GCLP).

Currently, radioimmunoassays (RIAs) are widely used and constitute a dominant role in PRA determination. On one hand, RIAs generally represent simple and highly sensitive radiometric methods. On the other hand, several detriments, apart from the inherent risks and costs in handling radioactive material, are mostly based on the short stability of the radioactive tracers due to radiolysis as well as challenges in disposal management [12]. This analytical effort may contribute to PRA not being regularly assessed in clinical research. More easily implemented ligand binding assays (LBAs) such as enzyme-linked immunosorbent assays (ELISAs) have not yet been embraced in the routine determination of PRA values in children, although their simplicity in handling might facilitate the sophisticated monitoring of PRA. The availability of such a method could be instrumental in minimising the previous knowledge gap of PRA in very young children suffering from HF.

The applicability of LBAs in paediatric research is often limited due to the required sample volume. Most of the commonly applied PRA RIAs (e.g. GammaCoat® Plasma Renin Activity 125I RIA Kit by DiaSorin Inc.) utilise high volumes of up to 1000 μL plasma. Such volumes exceed the sample volume required for the comprehensive analysis of pharmacokinetics/pharmacodynamics (PK/PD) as well as safety parameters for children [[13], [14], [15], [16], [17], [18]]. Furthermore, RIAs that can cope with small sample volumes of 50 μL–150 μL plasma frequently lack comprehensive validation, particularly in relation to U.S. Food and Drug Administration (FDA) regulatory guideline [19,20].

Although the detection of PRA by liquid chromatography coupled to mass spectrometry is also applied [21,22], high acquisition and maintenance costs, as well as the need for specialised staff, limits its routine application in academic-driven research consortiums.

The aim of the present research was the development and FDA compliant validation of a low-volume PRA ELISA applicable for GCLP settings in the context of the EU-funded Labeling of Enalapril in Neonates up to Adolescents (LENA) project (grant agreement n°602295).

## 2 Materials and methods

The quantitative characterisation of PRA by immunoassays (e.g. ELISA, RIA) is based on the determination of AngI values. The PRA can be calculated by comparing the AngI concentration in incubated human matrix with the AngI values of non-incubated matrix of the same sample. Measurement was accomplished by an in-house customised ELISA encompassing small volume applications. Units for PRA are expressed as μg/L/h, while units for AngI are expressed as μg/L. GCLP conformity was assured by a comprehensive validation of the developed assay using the FDA bioanalytical guideline, as well as further quality assurance tools (e.g. tracking software, audit trails, standard operation procedures, defined criteria for the validity of analytical runs, system suitability test, dual control principle, external audit, and ring test participation).

### 2.1 Materials

Generation buffer, phenylmethylsulfonyl fluoride (PMSF), rabbit anti-AngI antibody-coated microwell plates, AngI-biotin conjugate, streptavidin-horseradish peroxidase conjugate concentrate, assay buffer, wash buffer concentrate, tetramethylbenzidine (TMB) and 1 M sulphuric acid, as well as AngI calibration standards (CSs) and quality controls (QCs), were delivered by DRG Instruments GmbH (Springfield, USA). All CSs and QCs contained preservatives and emulated matrix (protein-based buffer) to mimic the effect of human matrix. HPLC gradient water was purchased from Fisher Scientific U.K. limited (Loughborough, United Kingdom). AngI stock solution, ethanol (>99.8% p.a.) and formic acid (≥98% p.a.) were obtained from Sigma-Aldrich (Steinheim, Germany). EIA buffer was purchased from Bertin Pharma (Montigny le Bretonneux, France). S-Monovettes (Sarstedt AG & CO, Nümbrecht, Germany) were used to obtain the required plasma samples from healthy volunteers. All pipetting steps were performed using calibrated Eppendorf Research pipettes in accordance with GCLP (Eppendorf, Hamburg, Germany).

### 2.2 Calibration standards

All seven non-zero CSs (0.2, 0.5, 1.5, 4, 10, 25, 60 μg/L) were utilised as a ready-to-use solution (analyte in artificial matrix) without further processing and contained AngI solutions. The PRA represents a calculated ratio of AngI values at 0 °C and 37 °C (incubated over a fixed period of time).

### 2.3 In-house customised assay procedure

#### 2.3.1 Pretreatment

Blood samples were thawed using a water bath (23 ± 3 °C). After adding 1 μL PMSF to 100 μL sample volume, the samples were vortexed for 20 s. Next, 10 μL generation buffer was added followed by a further 20 s of vortexing. The final mixture was separated into two aliquots. One aliquot was placed in an ice bath (0 °C), while the other aliquot incubated at 37 °C was put into a water bath. After 90 min, the generation process was stopped by placing the incubated aliquot in the ice bath for 5 min.

#### 2.3.2 Immunoassay

50 μL assay buffer was dispensed in every well of the microwell plate. Subsequently, 40 μL of CSs, blanks, QCs, as well as 0 °C and 37 °C aliquots were added, respectively. The microwell plate was incubated in the dark for 5 min at 500 rpm (24 °C) followed by a reduction of the shaking speed to 300 rpm for 15 min. Then, 80 μL AngI-biotin conjugate was added to each well and a second incubation for 5 min at 700 rpm in the dark (22 °C) was conducted. Afterwards, the shaking speed was reduced to 500 rpm and the incubation continued for 55 min. During the first automatic washing step (Tecan HydroFlex™, Männedorf, Switzerland), the contents of the wells were rinsed with five times 500 μL wash buffer and the residual liquids were subsequently removed thoroughly. Then, 170 μL streptavidin-horseradish peroxidase conjugate concentrate was added to all wells and incubated for 30 min at 22 °C and 500 rpm. Then, a second washing procedure (5 times 500 μL) was conducted and 170 μL TMB solution was added to each well, followed by 10 min of incubation at 500 rpm (22 °C) in the dark. The enzyme reaction was stopped by adding 60 μL sulphuric acid and additional mixing for 3 min at 350 rpm.

**Fig. 1:**
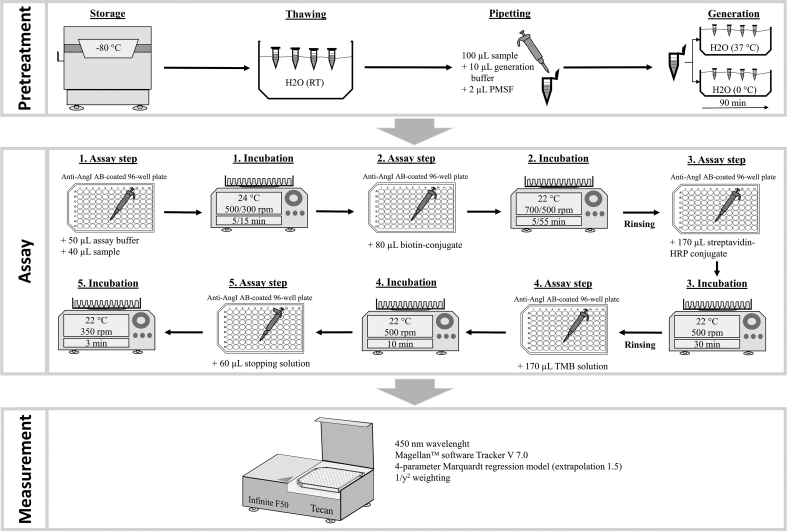
The modified plasma renin activity assay procedure. PMSF: phenylmethylsulfonyl fluoride; AB: antibody; HRP: horseradish peroxidase; TMB: tetramethylbenzidine; Rinsing: automatic washing step with 5 × 500 μL wash buffer and subsequent residual removal.

All incubation steps were performed by applying a ThermoMixer® by Eppendorf AG (Hamburg, Germany). The whole process is illustrated in Fig. 1.

### 2.4 Measurement of AngI and determination of PRA

The optical density (OD) measurement of AngI (450 nm) was performed using the absorbance reader infinite®F50 by Tecan (Männedorf, Switzerland). All calculations of concentration levels, as well as further statistical evaluations, were performed with the corresponding Magellan™ software Tracker V 7.0 based on the 4-parameter Marquardt regression model (extrapolation 1.5) with weighting 1/y^2^.

PRA was calculated using the following equation:

(1) $$PRA\;\left(\;\mu g/L/h\right)=\left(\frac{\left[AngI\;\left(37\;{}^{\circ}C\right)\right]-\left[AngI\;\left(0\;{}^{\circ}C\right)\right]}{1.5\;h}\right)*1.11$$

Equation (1): Calculation of plasma renin activity.

### 2.5 FDA based bioanalytical method validation

The method validation was performed based on the FDA bioanalytical guideline [9]. Due to the nature of the assay, the accuracy, precision, total error, calibration curve/linearity, and matrix effect was assessed by the direct evaluation of AngI values. For the validation parameters stability, parallelism, and the between-run precision of the whole process, the calculation of PRA values (Equation (1)) were conducted with the additional AngI generation step was included in the related validation runs.

#### 2.5.1 Calibration curve/linearity

The calibration curve consisted of seven non-zero CS levels (0.2, 0.5, 1.5, 4, 10, 25 and 60 μg/L). The calibration curve was evaluated in 12 independent runs on 12 different days. Each calibration curve was formed of at least six CS levels marked with a maximal relative error (RE) of ±20% (±25% at Lower Limit of Quantification (LLOQ)). Standards that did not comply with this criterion were excluded and the calibration curve was re-constituted. The linearity of each calibration curve was considered appropriate if the corresponding r-value was ≥0.995.

#### 2.5.2 Accuracy, precision, and total error

The accuracy, precision, and total error of the assay were investigated at five different concentration levels (0.2, 0.5, 1.5, 4, 10, 25 and 60 μg/L) in quintuplicate. Within-run accuracy and precision were investigated in one run, while between-run accuracy and precision were obtained in six individual runs on four different days. For the evaluation of within-run and between-run accuracy, the RE (as a measure for accuracy, Equation (2)) should not exceed ±20% (±25 for LLOQ). The coefficient of variation (CV) must be ≤ 20% (≤25% for LLOQ) for within-run and between-run precision. The total error was calculated as the sum of the absolute value of the RE and CV. The maximal limit of the total error for within-run and between-run investigation must be ≤ 30% (≤40% at LLOQ), following the recommendations of the FDA [10].

(2) $$Relative\;error\;\left(\%\right)=\left(\frac{measured\;AngI\;concentration-nominal\;AngI\;concentration}{nominal\;AngI\;concentration}\right)*100$$

Equation (2): Calculation of the relative error (%).

In addition, the between-run precision of PRA covering the whole process (sample pretreatment and determination) was assessed. Plasma samples of seven human sources were determined in triplicate at 15 different days concerning their PRA levels. The between-run precision (CV) per source must be ≤ 20%.

#### 2.5.3 Matrix effect

Matrix effects are defined as direct or indirect influences on the detector response caused by interacting substances within the sample.

The matrix effect was obtained by the comparison of native (plasma with endogenous levels of AngI only) and AngI-spiked samples of the same human source. Five different human sources were investigated, and samples were measured in triplicate. Exact final angiotensin I concentrations (obtained by the spiking of native samples with AngI working solution (1.05 μg/mL)) were dependent on the AngI concentration inherently present in native samples.

To minimise matrix dilution, the spiked solution did not exceed 10% of the total volume. The determined AngI values of four out of five sources should not deviate more than ±20% compared to the nominal AngI values.

#### 2.5.4 Parallelism

Plasma from one human source was spiked with AngI working solution (10.5 μg/mL) to reach high concentrations near the Upper Limit of Quantification (ULOQ). The following dilution steps were conducted: 1:2; 1:3; 1:4; and 1:5. All dilutions were performed using the highest concentration and the corresponding amount of EIA buffer to reach the intended dilution. In addition, a blank reduction was conducted by the investigation of native samples of the same source to determine the endogenous concentration of AngI still present in the native samples. The maximum deviation of the CV between the dilutions must not be more than 30%.

#### 2.5.5 Stability

Several stability experiments were conducted in the context of the FDA guideline to exclude an influence on analyte concentration due to limited stability during the study period, including sample preparation, and assay procedure. Analyte stability was ensured by the evaluation of freeze and thaw stability, short-term stability and long-term stability. For all experiments, freshly drawn plasma was aliquoted, snap-frozen and stored at −80 °C to mimic the same conditions as for study samples.

##### 2.5.5.1 Freeze and thaw stability

Freeze and thaw stability was examined by passing three freeze-thaw cycles of plasma from one human source. Snap-frozen human matrix was stored at −80 °C for a minimum of 24 h before being applied for the next freeze-thaw cycle. This procedure was repeated three times. After each freeze-thaw cycle, PRA was determined in triplicate and compared to the corresponding reference value (first value obtained). A maximum of ±20% RE of the mean value was allowed.

##### 2.5.5.2 Short-term stability

Short-term stability was investigated by the determination of PRA in human plasma for up to 2 h at room temperature (RT) and for up to 4 h at 0 °C to guarantee appropriate stability during the entire assay procedure.

For stability at RT, six aliquots were thawed and stored on ice. At time points 0, 0.25, 0.5, 1, 1.5 and 2 h, one aliquot was placed on the bench-top at RT. The sample for the reference value was continuously stored on ice. After the 2-h time point, all samples were measured simultaneously. Using the same method, the 4-h stability at 0 °C was assessed (time points of determination: 0, 2, 2.5, 3, 3.5 and 4 h (reference value)). All samples were measured in triplicate. The mean values of PRA should not deviate by more than ±20% from the mean PRA of the reference value.

##### 2.5.5.3 Long-term stability

The long-term stability was determined in seven human sources (three females, four males). Each human source was aliquoted and subsequently stored at −80 °C. Initial PRA reference values were obtained by calculating the mean of three independent analytical runs on two different days. Every 2 weeks, aliquots of each human source were thawed and measured in triplicate. The stability of AngI was proven if the deviation of the mean did not exceed ±20% of the reference value.

#### 2.5.6 Acceptance criteria of single analytical run

The assay was intended to be applied in the context of the LENA project which aims to generate data for a new marketing authorization. Therefore, recommendations provided by regulatory guidance for conduct of analytical runs were followed. As this within-run evaluation includes comprehensive monitoring of QCs as well as CSs, this regulatory guidance appeared to be more suitable for the project aim than the sole application of Westgard rules commonly used in routine laboratories.

In this regard, the acceptance criteria for a valid analytical run included CSs and QCs. The calibration curve in the PRA ELISA was established by no less than six ready-to-use CS. The CSs ranged from a concentration of 0.2 μg/L to 60 μg/L, containing CSs and a blank standard measured in duplicate. The back-calculated concentration of 75% of non-zero CSs including the LLOQ must not deviate by more than 25% and 20% for the calibrator at the LLOQ and the other calibrators, respectively. Exclusion of CSs was only acceptable if they failed the acceptance criteria or assignable causes. The validity of each run was assured by adding three QC levels (low, middle, high), at least measured in duplicate. The low and middle QC levels were directly delivered by DRG Instruments GmbH (Springfield, USA), while the high QC level was separately prepared in-house. A minimum of 50% of all samples per QC level must be within 20% of their nominal concentrations. Moreover, at least 67% of all QC samples must be within 20% of their respective nominal values.

### 2.6 Interlaboratory quality assessment

Besides the in-house validation procedure, the reliability of the ELISA was investigated by participation in an interlaboratory ring test in January 2018. To verify the accuracy, two samples of unknown concentration were obtained from the German Reference Institute of Bioanalytics (RfB) (Bonn, Germany) and analysed in duplicate. The measured values were back-reported and compared against 13 RIA based PRA reference methods to assess the general accuracy of the assay as well as its comparability to established RIA assays.

### 2.7 Application of the validated method

#### 2.7.1 Study samples

In order to demonstrate the applicability of the paediatric approach in the context of the EU-funded LENA project, example measurements were conducted using paediatric samples from three selected subjects suffering from HF (0.12, 0.37, and 0.69 years old). Blood samples were obtained before and 4 h after application of 0.25 mg enalapril maleate. All paediatric samples were collected in line with the Declaration of Helsinki. Written informed consent from the parent(s)/legal representatives and assent from the patient according to national legislation and as far as achievable from the child were obtained. Blood sampling was conducted by trained study staff following a specific time-monitored sampling procedure adapted to paediatric needs (e.g. use of microneedles) and parameter characteristics (e.g. limited time window between sampling, sample preparation and freezing) [23]. Each time point of the sampling process, as well as the behaviour and position of the neonate, was documented.

#### 2.7.2 Incurred sample reanalysis

CSs and QCs were constructed in an emulated matrix. Since potential differences between this matrix and paediatric matrix might impact the PRA determination by altering factors such as protein binding, back-conversion metabolites and the effects of co-medication incurred sample reanalysis (ISR) was conducted to confirm the reproducibility of measured study samples and the robustness of the assay [9]. ISR was calculated as follows (Equation (3)) [9,24]:

(3) $$Difference\;\left(\%\right)=\left(\frac{repeat\;value-original\;value}{mean}\right)*100$$

Equation (3): Calculation of the percentage difference between the original sample value and measured repeat.

In accordance with current international regulatory guidelines [9,10,24] the percentage difference was allowed to vary no more than ±30% of the mean value in 67% of all repeats. All ISR samples were obtained in the context of the LENA project.

## 3 Results

### 3.1 FDA based bioanalytical method validation

#### 3.1.1 Calibration curve/linearity

**Fig. 2:**
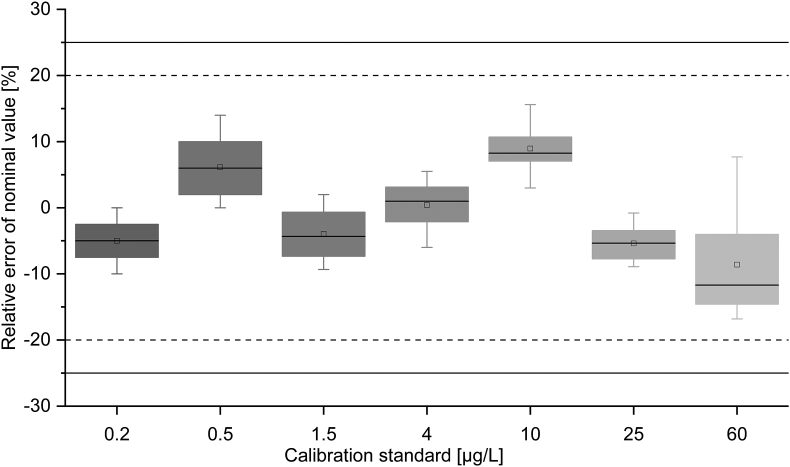
The relative error of all conducted calibration standards expressed as boxplots (12 runs). Solid lines: acceptance limits regarding U.S. Food and Drug Administration (FDA) at LLOQ; Dashed line: acceptance limits regarding FDA for the other calibrators. Box: 25–75%; : 1.5 interquartile range; : median; : mean.

In all twelve runs, the RE of all evaluated CSs was within ±20% of the nominal values (including LLOQ) and thus showed compliance with the FDA guideline (Fig. 2). All obtained r-values were ≥0.99884. Implementation of a 4-parameter Marquardt regression model (extrapolation 1.5) with weighting 1/y^2^ provided best fit for a calibration range of 0.2 μg/L to 60 μg/L.

#### 3.1.2 Accuracy, precision, and total error

Within-run accuracy over all five concentration levels showed a mean RE between −8.6% and +5.5% of the nominal values (mean of five determinations). Within-run precision varied between 2.3% and 11.1% (CV), while the total error ranged between 1.0 and 18.6%.

**Fig. 3:**
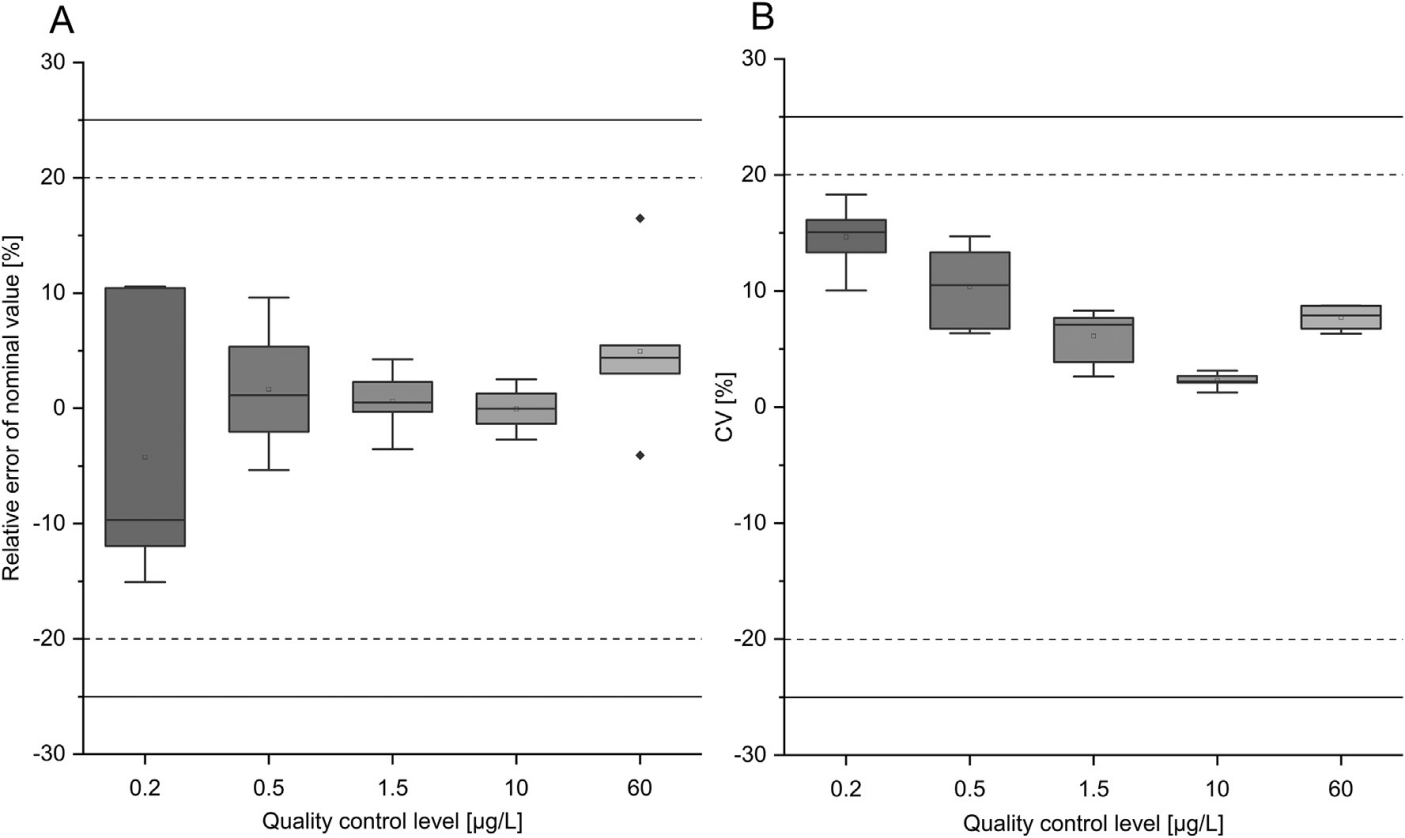
The relative error of between-run accuracy (A) and the coefficient of variation (CV) of between-run precision (B) for the plasma renin activity assay. Five individual quality control samples (including the Lower Limit of Quantification (LLOQ) were determined over six runs in quintuplicate. The six determined mean angiotensin I concentration levels for each of the six runs are shown as boxplots for every individual quality control level. Solid lines: acceptance limits regarding U.S. Food and Drug Administration (FDA) at LLOQ; Dashed line: acceptance limits regarding FDA for the other calibrators. Box: 25–75%; : 1.5 interquartile range; : median; : mean.

The RE for between-run accuracy was between 4.2% and 5.0%, while the between-run precision (CV) varied between 1.2% and 6.3%. Furthermore, the total error across all concentration levels was below 16.3%. For further details, please refer to Fig. 3.

In summary, the customised assay showed an appropriate accuracy and precision in the context of the applied bioanalytical guideline. All results for accuracy and precision were within the required limit of ±20% (±25% at LLOQ). Moreover, the total error for within-run and between-run investigations did not exceed the required limits of ±30% (±40% at LLOQ).

The determined mean PRA levels for the seven investigated sources amount to 2.85, 4.84, 0.79, 2.87, 1.96, 0.9, and 3.74 μg/l/h with a CV of 5.2%, 9.6%, 8.3%, 9.3%, 10.4%, 10.9%, and 9.0%, respectively. Therefore, the between-run precision of the whole process complied with the predefined limit of ≤20% (CV).

#### 3.1.3 Matrix effect

The obtained Ang I concentrations in the five evaluated human sources ranged from 23.9 μg/L to 29.0 μg/L AngI (mean of two replicates each). The percentage deviations to the nominal concentration varied between −20.4% and −3.3%. Since 80% of the investigated sources were well within the specifications (±20%) and one source showed borderline results (20.4%), no substantial matrix effect was claimed for the assay (Table 1).

**Table 1:** The results of investigated matrix effect in five human sources. Mean of two replicates each.

Source	Sex	Mean determined AngI concentration [μg/L]	Mean deviation of AngI concentration to nominal value [%]
1	♀	26.3	12.5
2	♀	29.0	3.3
3	♀	26.9	10.4
4	♀	27.6	8.2
5	♀	23.9	20.4

#### 3.1.4 Parallelism

All dilution steps were well within the calibration curve. The back-calculated mean AngI values were 35.1 μg/L for the undiluted sample, 32.5 μg/L for the 1:2 dilution, 29.5 μg/L for the 1:3 dilution, 27.7 μg/L for the 1:4 dilution, and 27.1 μg/L for the 1:5 dilution step. The inter-run precision (CV) was 11.1% over the five assay runs and thus did not exceed the limit of 30%.

#### 3.1.5 Stability

##### 3.1.5.1 Freeze-thaw stability

The RE of the stressed samples to the reference values showed a maximum of 11.3% after three freeze and thaw cycles. The within-run precision varied between 1.6 and 4.4% for PRA, while the between-run precision was 8.6% (CV). Since the deviations varied by no more than 20% from the reference values, accurate and precise sample analysis was not affected by the freeze-thaw cycles conducted.

##### 3.1.5.2 Short-term stability

All stability samples (2 ​h bench-top and 4 ​h at 0 ​°C) deviated by a maximum of +12.7% from the PRA reference value. Since PRA concentration level did not vary more than ±20%, the analyte can be considered stable at RT for at least 2 ​h and at least 4 ​h on ice.

##### 3.1.5.3 Long-term stability

**Fig. 4:**
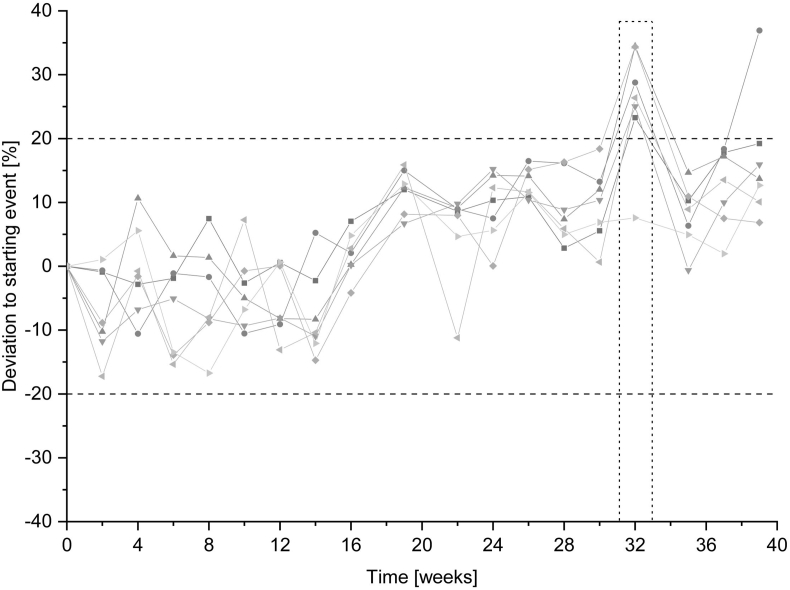
The percentage deviation of long-term stability for seven sources (indicated by the different symbols). Reference values of plasma renin activity were obtained by calculating the mean of three replicates of each source. Grey dashed lines indicate the maximum accepted deviation regarding U.S. Food and Drug Administration guidelines. Vertical dotted line marks the excluded run due to a processing error.

Long-term stability was proven for 20 runs over a time period of 260 days. However, the 18th run was excluded from the evaluation due to a processing error (deviation of water bath temperature). After the 20th run, one source exhibited a deviation of more than 20%, and the assessment of long-term stability was terminated. Overall, long-term stability for 260 days (ca. 37 weeks) was proven (Fig. 4).

### 3.2 Interlaboratory quality assessment

In addition to the in-house validation, an external verification by participation in a ring test executed by the RfB was successfully conducted. The ring test allowed for a comparison of the developed ELISA with established RIAs used by contract laboratories (for further details please refer to http://rfb.bio). As an ELISA reference value was lacking, the reported values were compared with RIA outcomes only. The latter allowed for a comparison of the easy-to-use ELISA to the more complex RIA setup.

**Fig. 5:**
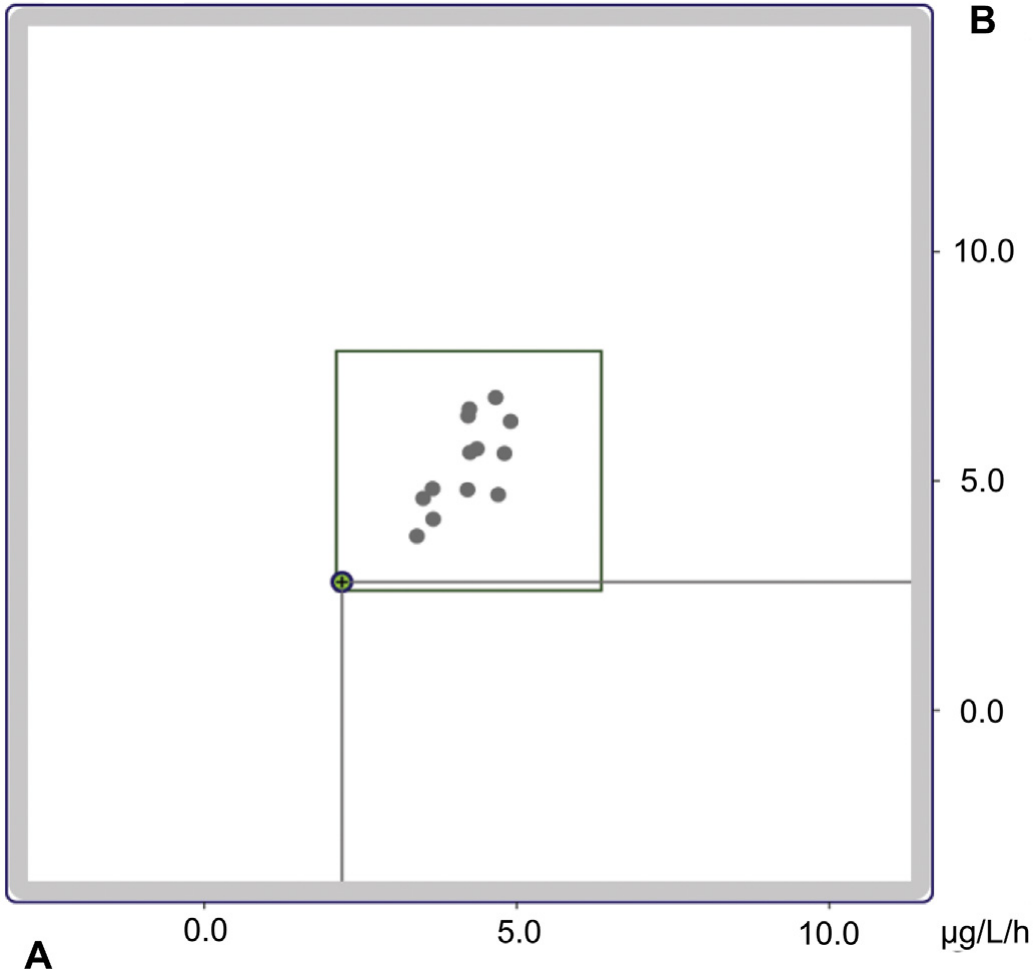
Youden plot for the results of interlaboratory quality assessment by the Reference Institute for Bioanalytics (Bonn, Germany). The x-axis represents sample A concentration while the y-axis represents sample B concentration. The marked circle indicates the reported values for sample A and sample B. Grey circles indicate other determination methods (radioimmunoassays). The rectangle indicates the maximum limits for a valid result.

The interlaboratory test samples (sample A and sample B) were measured a total of three times. The back-reported mean concentration values of the two unknown samples were 2.2 μg/L/h and 2.8 μg/L/h PRA. The RfB calculated the maximal accepted deviation based on all submitted assay results of the 14 participants. All other participating methods were conducted on the basis of RIAs. A valid result within the specification limits for both reported samples was achieved (Fig. 5).

By passing the ring test, the external quality assessment verified the reliability of the validated small-volume microassay.

### 3.3 Application of the validated method in paediatric patients

**Fig. 6:**
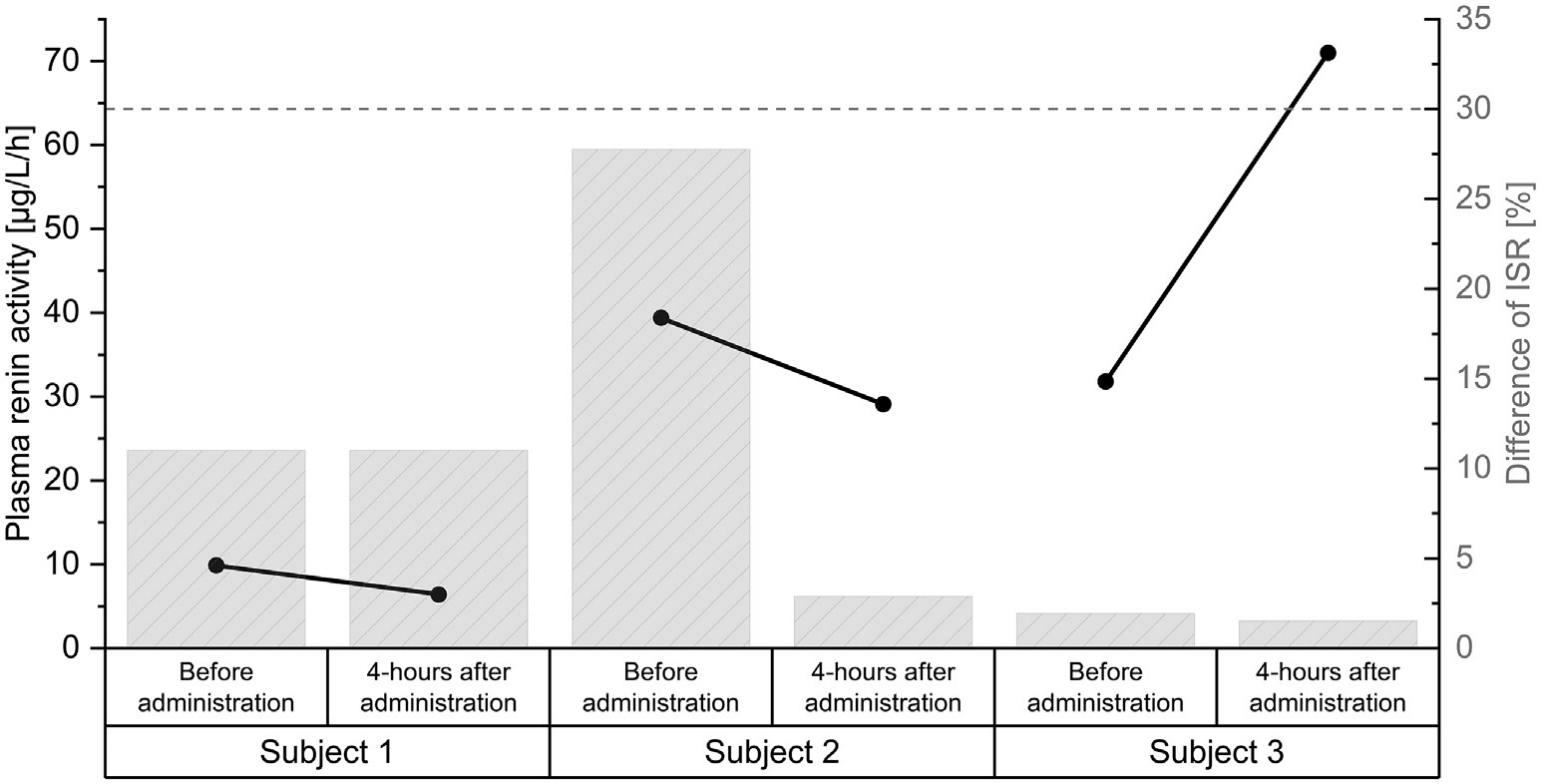
The plasma renin activity values (connected points/left y-axis) of three infants with congenital heart diseases before and 4 h after administration of enalapril maleate, as well as the relative deviation of each corresponding incurred sample reanalysis (ISR) (bars/right y-axis). Subject 1: 0.69 years of age; Subject 2: 0.12 years of age; Subject 3: 0.37 years of age; Dashed line: maximum accepted deviation for ISR.

Example paediatric study samples were successfully analysed to demonstrate the applicability of the assay using a real-life approach. Moreover, ISR was conducted to assess the comparability of the obtained data during the analytical runs within the study period, which is indispensable due to the unique data derived from the study population in the LENA project. All processes relevant for an accurate determination of humoral parameters (e.g. blood sampling, sample shipment, storage, and subsequent analysis) were conducted under GCLP conditions. The determined PRA values of the study samples and corresponding ISR are presented in Fig. 6.

#### 3.3.1 Study samples

The PRA values of three randomly selected neonates were determined before as well as 4 h after the administration of enalapril maleate. All six values were obtained in independent analytical runs. All conducted analytical runs were declared as valid in regard to the FDA acceptance criteria of an analytical run (2.5.6). Thus, the essential reduction of the applied sample volume from 500 μL to 100 μL enabled the decisive determination of PRA proving the applicability of the ELISA for paediatric trials.

#### 3.3.2 Incurred sample reanalysis

Since the CS/QC matrix used in the validation and the paediatric study matrix is not compulsively comparable, ISR samples were included in the analytical runs. In brief, six ISR samples were measured collectively with the study samples (one for each analytical run, conducted on different days).

The calculated difference for all six ISRs did not exceed ±30% and was thus well within the guideline limits, thereby substantiating the reproducibility of study samples under routine conditions. The paediatric-tailored microassay demonstrated the feasibility of obtaining high-quality data under GCLP conditions and complied with sophisticated guideline requirements. Therefore, the robustness of the microassay for the determination of PRA was proven, indicating the reliable determination of study samples in the context of the LENA project.

## 4 Discussion

The PRA ELISA presented here was successfully customised and validated regarding current international FDA bioanalytical guidelines. The assay serves the overall goal of application in vulnerable populations (e.g. elderly, severely diseased patients or children) by three properties. First, PRA determination was empowered by an easy-to-handle enzyme-linked immunosorbent assay instead of commonly applied radioimmunoassays. This could support the establishment of the assay in far more laboratories than the highly skilled laboratories with access to RIA. The latter facilitates wider data generation for PRA values of the RAAS in vulnerable population and could subsequently contribute to safer and more rational drug therapy. Second, the assay properties—especially the required blood volume—have been adapted for use in the aforementioned populations to reduce the burden of additional blood sampling in the context of simultaneous research to routine care. Third, the assay was validated according to current FDA bioanalytical guideline to ensure reliable data generation and its application in a GCLP-compliant environment.

The recently published EMA concept paper on guidelines for the investigation of medicinal products in term and preterm neonates demands a greater focus on organ and enzyme system maturation differences across several paediatric age groups [25]. However, these differences and their pharmacodynamic effects can only be sophisticatedly investigated if appropriate microassays are applied. The comprehensive understanding of adult RAAS has resulted in over 20 drugs being approved for HF in adults; however, only a few approved drugs are available to treat HF in all paediatric age groups. New potential target structures within the RAAS are continuously identified and provide further insight into the adult RAAS [26], while the intervention on these humoral parameters remains unknown in children. Therefore, urgently fostered research on PRA and the maturating RAAS can only be accomplished by the establishment of customised assays. Against this background, the ELISA PRA assay was customised and characterised by a five-fold reduction in required plasma volume. Based on ethical recommendations in clinical trials [11], the total blood loss for routine care and research in neonates must not exceed 3% over 4 weeks (1.2 mL–1.4 mL plasma per kg body weight). Even LBAs, which were used for paediatric investigation of PRA (e.g. GammaCoat® Plasma Renin Activity 125I RIA Kit by DiaSorin Inc [13]) require high sample volumes of 250 μL–1000 μL for a single determination; therefore, this limits the amount of practicable analysis (e.g. determination of multiple PK/PD parameters beside investigation of safety samples) [14,15]. Although other small-volume RIA assays (50 μL up to 150 μL for PRA determination) appropriate for investigation in children exist [19,20], a validation regarding international guidelines has not been demonstrated and subsequently does not allow for application in clinical trials. The substantial reduction in sample volume (500 μL–100 μL) of the presented ELISA—without restricting the calibration range (0.2 μg/L to 60 μg/L) [27]— also enables the examination in the diseased paediatric population (e.g. children suffering from HF) [13,[16], [17], [18],^28^].

Moreover, the ELISA was successfully validated concerning the FDA bioanalytical guideline. This verification of quality is important in the context of applying the assay in a regulatory environment—a factor that most published assays lack [14,15,19,20]. In particular, clinical studies in paediatrics are usually only performed once owing to ethical constraints. Therefore, it is of utmost importance that the generated bioanalytical data is of high quality and reliable as it will be used for decision making related to paediatric drug therapy. In addition to the successful in-house validation procedure of the presented ELISA, participation in external independent quality assurance (ring trial) confirmed the accurate performance of the customised ELISA. Furthermore, the comparability of the ELISA results with routinely applied RIA was assessed within this external quality assurance.

The outcome of this comparison indicated non-inferiority regarding the reported reference RIAs, thus highlighting the usefulness of the presented ELISA for routine application. Besides the applied analytical technique, the sample matrix used is also known to influence reliable determination. Protein binding, sample inhomogeneity, known or unknown metabolites and co-medication might affect accurate sample determination. For example, such effects can be prone to maturing organisms and therefore require a corresponding investigation. Therefore, the applicability of the assay in paediatric research (e.g. the LENA project) was outlined by the random determination of six paediatric samples with corresponding ISR in different analytical runs. The conducted ISR confirmed the accurate performance of the assay in a real-application approach and the reproducibility of unknown sample results obtained in different runs. In conclusion, the in-house validation, external quality assurance and applicability demonstrated the assay’s reliability, fit-for-purpose and usefulness for accurate determination of PRA in vulnerable populations.

Notably, small-volume ELISAs are not only suitable for examination in children and can also unburden severely diseased adults. The total volume of blood withdrawal and the number of blood draws in critically ill patients is related to increased consumption of blood transfusions, higher incidence of anaemia and illness severity [29]. In addition, *Ullman* et al. reported that anaemia occurs in 95% of all patients admitted to intensive care units at day three, which increases the risk of severe events in critically ill patients [30]. The reduced required blood volume could also facilitate the ongoing monitoring of patients and contribute to avoiding additional burdens due to increased blood loss. An application of GCLP-compliant small-volume bioanalytical assays can thus bring a decisive advantage, even in an adult population.

In brief, an FDA-compliant validation was successfully accomplished for the developed microassay, enabling a reliable investigation of PRA in pivotal trials. In addition, this downscaled ELISA is applicable for paediatric trials and successfully copes with limitations in blood volume, providing a valuable alternative to commonly used RIAs.

## 5 Conclusion

The development and validation of a fit-for-purpose PRA ELISA were accomplished for paediatric application, indicating non-inferiority to commonly used RIAs. The FDA-compliant PRA assay is able to accurately and precisely quantify PRA values in 100 μL plasma and is applicable for GCLP-compliant clinical studies, thus enabling sophisticated investigations in children within paediatric clinical studies.

## Funding

The research leading to these results has received funding from European Union Seventh Framework Programme (FP7/2007-2013) under grant agreement n°602295 (LENA).

## Declaration of competing interest

None.
